# QuEChERS extraction coupled to GC-MS for a fast determination of polychlorinated biphenyls in breast milk from Polish women

**DOI:** 10.1007/s11356-019-06201-y

**Published:** 2019-08-26

**Authors:** Martyna Pajewska-Szmyt, Elena Sinkiewicz-Darol, Urszula Bernatowicz-Łojko, Tomasz Kowalkowski, Renata Gadzała-Kopciuch, Bogusław Buszewski

**Affiliations:** 1 Department of Environmental Chemistry and Bioanalytics, Faculty of Chemistry, Nicolaus Copernicus University in Toruń, 7 Gagarin St., 87-100 Toruń, Poland; 2 Interdisciplinary Centre for Modern Technologies, Nicolaus Copernicus University, 4 Wileńska St., 87-100 Toruń, Poland; 3 Ludwik Rydygier Provincial Polyclinic Hospital in Toruń, Human Milk Bank, Św. Józefa 53-59, 87-100 Toruń, Poland; 4 Human Milk Bank Foundation, 128J Podkowy St., 04-937 Warsaw, Poland

**Keywords:** Polychlorinated biphenyls, Breast milk, QuEChERS method, Gas chromatography, Mass spectrometry

## Abstract

Polychlorinated biphenyls (PCBs) in breast milk has been determined. Therefore, it was necessary to develop and adapt an analytical method to analyze PCB compounds. The whole procedure was applied to 31 breast milk samples, which were collected from Polish mothers. The QuEChERS method was optimized as a fast and cheap sample preparation method. The procedure allowed us to obtain recovery values between 96.46% and 119.98% with acceptable relative standard deviations (3.36–12.71%). Gas chromatography with mass spectrometry (GC-MS) was used for final determination. The method was validated using parameters such as linearity, limit of detection and quantification, intra-day precision, and reproducibility. The mean concentration of *∑*iPCBs in this study was 30.94 ng/g of lipid. Assigned daily intake of PCBs was lower than the tolerable daily intake, which shows that the analyzed milk is safe to the infants. However, the monitoring of PCBs in milk is still important, and the QuEChERS method with GC-MS can be an effective tool for tracking organic impurities in breast milk.

**Electronic supplementary material:**

The online version of this article (10.1007/s11356-019-06201-y) contains supplementary material, which is available to authorized users.

## Introduction

The presence of environmental pollutants is very dangerous for living organisms as these impurities can cause significant health problems. Organic contaminants such as polychlorinated biphenyls (PCBs) belong to the group of halogenated aromatic hydrocarbons. These compounds are made up of two phenyl rings with attached chlorine atoms (between 1 and 10) (Andersson et al. 1997). Polychlorinated biphenyls are divided into three groups: poly-ortho, mono-ortho, and non-ortho substituted PCBs. The last two groups can bind to the Ah receptor and their toxicity is similar to that of dioxin (Ulaszewska et al. 2011).

PCBs were used in electrical insulation, hydraulic fluids, plasticizers, and additives in glue and copy paper for over 50 years. Their commercial production was banned in the late 1970s (Erickson and Kaley II 2011). However, human exposure continues as a consequence of the widespread past use of these chemicals (Černá et al. 2012). Moreover, polychlorinated biphenyls have lipophilic properties and are resistant to degradation process (long half-lives). If these compounds penetrate into a water body, they bioaccumulate in fish (Bu et al. 2015; Marin et al. 2011). The solubility of PCBs in water is low, but they are mostly suspended in organic matter, which for aquatic organisms is the most common source of sustenance. Thus, consumption of fish products is risky as consumers may be exposed to organic compounds present in such food.

People can be exposed to PCBs in many ways, such as dietary intake, dermal contact, and inhalation. These toxicants can influence various body organs as well as the reproductive, nervous, and endocrine systems, for example, the production of thyroid hormones (Soechitram et al. 2017). Unfortunately, one of the ways of eliminating these compounds from the body is lactation.

Human milk has high lipid content (3.5 to 4.5/100 g in mature milk), and polychlorinated biphenyls are binding with fats and are transferred to infants through breastfeeding (Delplanque et al. 2015). WHO recommends human milk as a non-invasive matrix to estimate the level of lipophilic chemicals. Some countries systematically conduct pollution monitoring, most notably the Czech Republic (Černá et al. 2010), Slovakia (Čechová et al. 2017), and China (Deng et al. 2012; Zhang et al. 2017). The first research regarding PCB in milk of Polish women was conducted by Czaja and co-workers (Czaja et al. 1997); the majority of later publications present research on breast milk from Wielkopolska region (Jaraczewska et al. 2006; Škrbić et al. 2010; Szyrwińska and Lulek 2007), and the last paper published in 2014 concerned samples from central Poland (years 2008–2010) (Kamińska et al. 2014).

Prenatal and postnatal exposure of infants to PCBs can be associated with health problems. Vulnerable newborns are particularly sensitive due to incomplete development of the immune, respiratory, and nervous systems. These environmental toxicants can be linked with such illnesses as asthma or allergy (Gascon et al. 2013; Lignell et al. 2013). The adverse effects of toxic compounds may also include the thyroid system of infants (Maervoet et al. 2007). Epidemiological studies performed by Verner and co-workers show correlation between exposure to PCB 153 and children problem with anxiety and attention deficits (Verner et al. 2015). There are also reports on the possible relationship between persistent organic pollutions and risk of attention-deficit hyperactivity disorder (ADHD) (Caspersen et al. 2016; Lenters et al. 2019). The research carried out by Norwegian scientists shows also the impact of environmental pollution on microbial flora and functions of infant gut (Iszatt et al. 2019). Therefore, monitoring of milk for the presence of these compounds is essential for exposure assessment of both mothers and infants.

The detection method used for polychlorinated biphenyls is gas chromatography, most often combined with electron capture detector or mass spectrometry (Deng et al. 2012; Hassine et al. 2012). A major step in PCB determination is sample preparation. Human milk is a very heterogeneous matrix, which needs a specific method of sample cleanup. Classical liquid-liquid extraction (LLE) (Bencko et al. 2004; Hassine et al. 2012) and solid-phase extraction (SPE) (Lin et al. 2016) are typical methods used for isolation of PCBs from samples. To minimize use of hazardous solvents as well as the cost and time of sample preparation, it is important to improve this stage of analysis, also with a view to obtaining better results for specific types of analytes and matrices.

In 2003, a method that was quick, easy, cheap, effective, rugged, and safe (hence the acronym QuEChERS) was developed by Anastassiades and co-workers (Anastassiades et al. 2003) for pesticide residue in fruit and vegetables. This technique has been reported in an increasing number of various applications. Generally, the QuEChERS approach can be divided into two stages. The first is extraction using organic solvents and inorganic salts (MgSO_4_, NaCl, or/and buffering salts) to separate water-organic phase, followed by dispersive solid-phase extraction (dSPE) step, which is used to remove interferents such as pigments, sugars, and organic and fatty acids from the extract (Morrison et al. 2016). This method can be easily modified by using various combinations (amount, type, ratio) of solvents, sorbents, or salts, and it is dependent on the analyte and the matrix. The crucial issue is to efficiently remove lipids because they reduce sensitivity and reproducibility of instrumental analysis (Baduel et al. 2015). The QuEChERS method has also been applied to isolating PCBs from fish tissue (Chamkasem et al. 2016; Han et al. 2016; Morrison et al. 2016; Norli et al. 2011; Peng et al. 2015), mussel (Madureira et al. 2014), meat (Kuzukiran and Filazi 2016), and honey (Al-Alam et al. 2017). As regards breast milk investigations, three papers were found in literature (Asamoah et al. 2018; Baduel et al. 2015; Luzardo et al. 2013). The list of examples of studies with the QuEChERS approach for PCB is presented in Table S1.

The present study aimed to adapt the QuEChERS method for breast milk samples in order to analyze PCB compounds. A particular GC-MS method was developed to analyze seven target PCBs (28, 52, 101, 118, 138, 153, 180) which were selected because these non-dioxin-like PCBs are most frequently found in the environment and thus are representative of the whole PCB group (Baars et al. 2004). Moreover, this study had two objectives: the first was to propose a fast method which can be used for monitoring pollutants in human samples, and the second was to apply the developed procedure to analysis of PCB contents in breast milk from Polish mothers. The second step was important considering lack of monitoring in Poland, especially in Kuyavian-Pomeranian region.

## Materials and method

### Samples

Thirty-one breast milk samples were obtained from mothers living mostly in Kuyavian-Pomeranian region. Sampling was done between July and December 2017. The women donated their breast milk between 1 week and 11 months after giving birth. Breast milk was manually transferred into clean 100-mL bottles. It is important to note that the samples donated to the milk bank were in most cases the product of overabundant milk supply in mothers, and the newborns were not at risk of being underfed; also, the study used volunteer participants. The following information was provided with each sample: mother’s age, date of sampling, and number of pregnancies and deliveries. Moreover, immediately after collection, each breast milk sample was analyzed in the hospital laboratory for the following parameters: lipid content, total protein, nutritional protein, carbohydrates, dry weight, and energy value (Table S2). The milk was stored at − 20 °C prior to analysis.

### Reagents and solvents

The following reagents were used in the study: deionized water (Milli-Q Reagent Water, < 10 MΩ cm^−1^ resistivity, Merck, Millipore); acetonitrile for HPLC, n-hexane for GC (Sigma-Aldrich, Poznan, Poland); sulfuric acid; salts – anhydrous magnesium sulfate (99.5% purity), sodium chloride (99.9% purity) (Avantor Performance Materials Poland S.A., Gliwice), sodium citrate monobasic (99.5% purity) (Sigma-Aldrich, Poznan, Poland); sorbents – primary and secondary amines – Bondesil PSA, 40 μm (Labstore, Warsaw, Poland), Bakerbond octadecyl (C18, 40 μm, 60 Å), and silica gel (40 μm, 60 Å) (S. Witko, Lodz, Poland). Acetonitrile was saturated with n-hexane (acetonitrile:n-hexane 1:1 (v/v) added to a separation funnel and shaken for 1 min); then, the solvents were allowed to separate phases, and the lower layer was used as extraction solvent. The concentration of the indicators of polychlorined biphenyl standard solutions (Dr. Ehrenstorfer, Germany) was 100 ng/mL. Solutions with lower concentration were produced by diluting the working solutions in n-hexane solvent to obtain concentrations between 2 ng/mL and 11 ng/mL.

### Instrumentation

GC-MS analysis was performed using Agilent Technologies 6890N (for GC) and 5975 (for MS) in EI mode. The temperatures of the ion source and interface were 300 °C and 280 °C, respectively. Chromatographic separation was conducted on a Phenomenex capillary column ZB-5MS (30 m × 0.25 mm × 0.25 μm). The carrier gas flow rate was 1.1 mL/min. The injection temperature was 265 °C. The volume of injected samples was 1 μL. The temperatures were programmed as follows: the initial oven temperature of 60 °C was maintained for 1 min, ramped at 20 °C/min to 170 °C, maintained for 0.30 min, and then raised by 10 °C/min to 310 °C with a 1.20-min hold time. In every run, the selected ion monitoring (SIM) mode was used (Table 1).

**Table 1:** Retention time, SIM mode, and recovery calculated for three concentration levels and matrix effect for selected PCBs

PCB congener	IUPAC name	Retention time (min)	SIM ions	Recovery (%) (RSD)			Matrix effect (%) (RSD)
				10 (ng/mL)	5 (ng/mL)	2 (ng/mL)	
28	2,4,4′- Trichlorobiphenyl	12.118	150.05/186.00/255.90/257.90	96.46 (8.33)	110.61 (9.54)	110.73 (10.36)	3.50 (1.40)
52	2,2′,5,5′-Tetrachlorobiphenyl	12.728	150.05/220.00/254.85/291.90	102.51 (4.86)	116.09 (7.18)	104.44 (8.82)	7.53 (1.14)
101	2,2′,4,5,5′-Pentachlorobiphenyl	14.331	184.00/253.90/325.80	114.04 (8.66)	105.79 (12.71)	117.76 (3.69)	9.68 (0.73)
118	2,3′,4,4′,5′-Pentachlorobiphenyl	15.488	183.90/253.90/255.90/325.80	107.12 (8.76)	111.82 (8.96)	115.03 (8.11)	11.30 (0.31)
153	2,2′,3,4,4′,5′-Hexachlorobiphenyl	16.387	217.90/289.90/359.80	103.61 (7.19)	105.76 (8.70)	119.98 (3.45)	8.34 (1.24)
138	2,2′,4,4′,5,5′-Hexachlorobiphenyl	15.877	217.95/289.90/359.80	103.98 (7.13)	107.86 (8.62)	103.10 (8.45)	6.77 (0.45)
180	2,2′,3,4,4′,5,5′-Heptachlorobiphenyl	17.580	251.95/323.80/393.75	101.53 (4.46)	102.64 (6.11)	102.78 (7.91)	1.53 (2.45)

### QuEChERS method

The proposed method is based on the procedure described by Luzardo et al. (2013) in which we introduced minor modifications. Five milliliters of milk was transferred into a 50-mL Falcon tube. Next, 5 mL of water was added and the Falcon content was vortexed. During mixing, acetonitrile saturated in n-hexane (10 mL) was added. The mixture was allowed to stand for 30 min and every 10 min, it was vortexed. The salts of anhydrous magnesium sulfate (4.0 g), sodium chlorine (1.0 g), sodium citrate monobasic (0.5 g), and disodium citrate (1.0 g) were added to the content of the tube. The mixture was shaken for 1 min. After centrifugation (5000 rpm, 5 min), the upper phase was transferred to a glass vial and the residue in the Falcon tube was co-extracted with 5 mL of acetonitrile saturated in n-hexane. After the Falcon tube was shaken for 1 min, centrifuging was performed and the upper phase was transferred again to a glass vial. Two extracts were combined and the whole content was transferred to another Falcon tube which contained magnesium sulfate (0.9 g) and primary and secondary amines (0.3 g). The mixture was shaken for 1 min and centrifuged (5000 rpm, 5 min). The extract was evaporated in a water bath (40 °C) to dryness under gentle nitrogen stream (low gas pressure 3–4 bar). The residue was then dissolved in 500 μL n-hexane, and 250 μL of concentrated sulfuric acid was added. The sample was vortexed and centrifuged (3000 rpm, 10 min). The hexane layer was transferred to a vial, ready for GC-MS analysis.

### Validation

#### Linearity, limit of detection, limit of quantification, and intra- and inter-day precision

According to the International Conference on Harmonization (ICH) guidelines on validation of analytical procedures (ICH 2005), selected validation parameters were determined, such as linearity, limit of detection, and limit of quantification as well as intra-day and inter-day precision.

The calibration curves were determined by measuring seven concentrations of the indicator polychlorinated biphenyls in five replicates (2, 3, 4, 5, 7, 9, 11 ng/mL). The limit of detection was determined as the area (S) of the analyte concentration of three times the background noise (N) (LOD = 3S/N), and the limit of quantification as a peak signal of ten times the background noise from the chromatogram (LOQ = 10S/N). Intra-day and inter-day precision was determined from analyses done on the same day (intra-day repeatability) and from analyses done on different days (reproducibility); they were shown as relative standard deviation.

To determine recovery, milk was spiked with three concentrations of PCB standard solution (2, 5, 10 ng/mL), and recovery was calculated as the ratio of the area of the contaminated sample to the area of the standard solution.

#### Matrix effect

To calculate the matrix effect of the proposed method, the following formula was used:

1 $$ \mathrm{ME}\%=\left(\frac{x_2-{x}_1}{x_1}\right)\bullet 100\% $$

where *x*_1_ is the mean area of the standard solution and *x*_2_ is the mean of the PCB standard solutions in blank extract. Using this formula makes it possible to determine matrix effect, which can be the response of the detector signal (Pizzutti et al. 2009).

### Quality control of samples

Standard solutions of the investigated compounds representing low, middle, and high concentrations from the range of the calibration curve were included in each run. Furthermore, in each batch of samples, a blank sample (milk without PCBs) and contaminated milk (milk spiked with standard solutions of PCBs) were included. The peaks were identified as target compounds when the signal was higher than the limit of quantification (LOQ), and the retention time in comparison to the standard did not exceed ± 0.05 min.

### Daily intake

The average weight of an infant is 5 kg, and in a day, a child consumes on average 700 g of milk. Daily intake (DI) of indicator polychlorinated biphenyls was calculated according to the following formula (Asamoah et al. 2018; Klinčić et al. 2016; Van Oostdam et al. 1999):

2 $$ \mathrm{DI}=\frac{C_{\mathrm{milk}}\bullet 700\mathrm{g}\frac{\mathrm{milk}}{\mathrm{day}}\bullet {C}_{\mathrm{lipid}}/100}{5\ \mathrm{kg}\ \mathrm{body}\ \mathrm{weight}} $$

where *C*_milk_ is the concentration of *∑*iPCB (μg/g lipid weight), and *C*_lipid_ is the lipid content in milk (%).

### Data analysis

Multivariate statistical methods were applied to evaluate similarities and differences between the collected milk samples. The content of seven PCBs in samples was used as a dataset. Cluster analysis (CA) and factor analysis were performed. Euclidean distances were calculated to create distance matrix. Both tree clustering method based on Ward’s agglomeration rules and two-way clustering were applied. Factor analysis was based on principal component with varimax rotation. Three latent factor axes were derived, facilitating differentiation of milk samples. Statistica DataMiner 7.0 (Statsoft, Poland) was used to calculate the relevant information.

## Results and discussion

### Optimization of extraction and cleanup stage

The first attempt—when Luzardo’s QuEChERS procedure was applied (Luzardo et al. 2013) for sample extraction—was unsuccessful because numerous interferences appeared in the extraction solution, which significantly decreased the limit of detection. Moreover, it was possible to identify only three out of seven PCBs. Furthermore, unpurified extract of milk with lipids could damage the chromatographic column. It was necessary to check the extraction steps and make changes in the procedure in order to obtain satisfactory results. The following solvents used for extraction have been tested: acetonitrile saturated with n-hexane, acetonitrile, and hexane:acetone 1:1. In the last case, the extract contained a large amount of co-extracted compounds. Conversely, by using acetonitrile, co-extraction of interferents was minimized. Moreover, extraction with acetonitrile followed by addition of salt improved separation of water-organic phase (Morrison et al. 2016). The highest efficiency for PCB extraction was observed for acetonitrile saturated in n-hexane, so this solvent was used in further studies.

The most important step was the cleanup procedure. In the QuEChERS method, dispersive solid-phase extraction is used to purify the extract. The purpose of the used sorbent is to absorb interfering substances and keep the PCBs in the extract. Luzardo et al. (2013) proposed to use MgSO_4_ (0.9 g) and PSA sorbent (0.5 g). We tested also silica gel and C18 sorbents (using standard solutions) to check the retention of PCBs on these sorbents. It was found that silica gel stops the polychlorinated biphenyls with lower number of attached chlorine atoms (content of PCBs in extract: PCB28 1.88%; PCB52 14.47%; PCB101 38.12%; PCB118 60.45%; PCB153 73.15%; PCB138 72.88%; and PCB180 74.50%). In the case of C18, the results show that this sorbent did not absorb PCB (in standard solutions) and the recoveries ranged from 78 to 120% for individual PCBs when only standard solutions (without milk) were used. The 0.25 g of C18 sorbent was included at the dSPE stage to modify the Luzardo et al. (2013) procedure and to check whether this could improve the recovery and cleanup of the sample. Unfortunately, in matrices with standard solutions, the recovery was high enough only for PCB101 (71.18% on average). For the rest of the PCBs, the recovery was below 50%. As a result of the presence of interfering substances (e.g., fats) in such a real sample as milk, the PCB content in the extract was not high. Additionally, purity of the sample remained unsatisfactory. The reason for this is that C18 sorbent removes non-polar substances such as lipids. Considering the fact that PCBs have a tendency to bind with fat and C18 sorbent is able to retain lipophilic residues, the recovery as a consequence is low (Chamkasem et al. 2016). Moreover, if water is not completely removed, the majority of lipophilic compounds are lost instantly (Berendsen et al. 2013; Molina-Ruiz et al. 2015). Therefore, we decided not to utilize silica gel or octadecyl sorbent at the cleanup stage. The PSA sorbent used by Luzardo and co-workers (Luzardo et al. 2013) was the best option. PSA, which has a primary and a secondary amine, binds matrix co-extractives such as sugars and fatty acids; however, PSA offers more effective isolation of investigated polychlorinated biphenyls from lipids. Summarizing, PSA allows separation of PCBs from fats; consequently, co-extracted interferents are adsorbed by the sorbents while analytes remain in the extract. In our procedure, the necessary amount of PSA sorbent was reduced to 0.3 g. Furthermore, to minimize the amount of co-extracted lipids, after the residue had been dissolved in hexane, concentrated sulfuric acid was added to the content of the sample; this approach was used for example by Rojas-Squella et al. (2013). Sulfuric acid causes destruction of lipids and this way is an improvement over lipid removal by freezing. The sample was vortexed and centrifuged; after which, the extract was transferred to a vial for GC-MS analysis. The influence of sulfuric acid addition is presented in Figure S1. Consequently, the whole procedure allowed us to obtain high recovery values (96.46–119.8%) with acceptable relative standard deviations (3.69–12.71%) (Table 1).

Moreover, a review of the literature was conducted to compare the proposed method with others. The most frequently used methods are traditional techniques such as accelerate solvent extraction (ASE) (Deng et al. 2012; Ottonello et al. 2014; Vigh et al. 2013), liquid-liquid extraction (LLE) (Chovancová et al. 2011; Colles et al. 2008; Hassine et al. 2012), Soxhlet extraction (Zhao et al. 2008), or solid-phase extraction (SPE) (Lin et al. 2013; Salihovic et al. 2012; Zhang and Rhind 2011). Unfortunately, the problem with comparing these studies is lack of information about such issues as limit of detection/quantification, recovery, or precision. Most of the research investigating human milk are cross-sectional studies, where the selected method of sample preparation and detection is only a tool in the assessment of PCB content. The procedures are based on standard methods or at least those that have already been developed and validated. As such publications focus on longitudinal studies and correlations with different life factors, presentation of validation parameters is omitted. However, it can be concluded from the collected data that the limit of detection of PCBs in milk with the QuEChERS method (1.05–2.39 ng/g) was lower than with LLE (5.0 ng/g or 20 ng/g) (Chovancová et al. 2011; Colles et al. 2008). In a study conducted by Hassine and co-workers (Hassine et al. 2012), the LOD was lower but the recovery was smaller than what we achieved in this study. Comparing this method with other examples that used different matrices, it can be noted that better reproducibility has been achieved (RSD < 11%). Furthermore, the QuEChERS technique allows shorter sample preparation time compared with traditional methods. In addition, if traditional methods are used for such a matrix, it is necessary to include a cleanup procedure with silica gel or aluminum oxide columns, which is time-consuming and labor-intensive.

### Method validation

The results of the GC-MS analyses were validated. Linearity was satisfactory in all cases, as demonstrated by high correlation coefficients (0.995–0.999). The limit of quantification for all the analyzed polychlorinated was at the range of 0.74–1.65 ng/mL for particular PCBs (limit of detection, 0.22–0.58 ng/mL). The method showed acceptable intra-day precision (RSD 4.43–13.24%) and reproducibility (RSD 4.71–10.45%). Furthermore, the matrix effect (determined based on Eq. 1) was below 12%, which is satisfactory because the matrix effect for PCBs is insignificant or irrelevant (Table 1).

Final recovery (the ratio of the area of the spiked samples to the area of the standard) was at a satisfactory level for all the studied compounds (96.46–119.98%), with acceptable relative standard deviation (3.69–12.71%). The obtained data are summarized in Table 1. The above results confirm that the proposed method meets the validation criteria and can be applied successfully in further research.

### Analysis of breast milk samples

The mean concentration of *∑*iPCBs in this study was 30.94 ng/g of lipid (range <LOQ–119.87 ng/g of lipid). In comparison to previous research in Poland (done in most cases in Wielkopolska) (Table 2), the obtained level is much lower than that provided by the research conducted in Poznań vicinity (Szyrwińska and Lulek 2007). Our samples were collected in 2017, 10 years after the last study in Poznan was carried out. Due to this gap, it is difficult to compare both studies. Furthermore, higher population density favors higher PCB content (Soechitram et al. 2017), and the Poznań region has higher population density than Toruń (2067 people/km^2^ and 1750 people/km^2^_,_ respectively). Poznań is also one of the largest economic centers in Poland. Similar reasons can also influence the comparison of our results with those from Warsaw (mean *∑*PCBs = 151 ng/g). The mean content of PCBs measured in milk samples from Toruń is lower than that in other European countries including Slovakia, the Netherlands, Norway, and Russia. However, compared with other continents, the level of polychlorinated biphenyls in breast milk is higher (Table 2).

**Table 2:** Examples of investigation studies on PCB in breast milk from different countries

Country	Sampling year	Number of samples	*∑*iPCB mean (ng/g of lipid)	*∑*iPCB Range (ng/g of lipid)	Reference
Poland (Toruń, Kujawsko-Pomorskie)	2017	30	30.94	<LOQ-119.87	This study
Poland (Poznań, Wielkopolskie)	2000–2001	27	114.8	29.9–485.9	Szyrwińska and Lulek (2007)
*Poland (Łódź/Łask, Łódzkie)	2008–2010	40	–	dl-PCBs 0.0015–0.019	Kamińska et al. (2014)
Wielkopolska	2004	22	*∑*PCB (15) 153	63–413	Jaraczewska et al. (2006)
Wielkopolska	2000–2001	12	77.6	–	Lulek et al. (2002)
Warszawa	2002–2005	28	*∑*PCB(8) 151	–	Hernik et al. (2011)
Slovakia	2010–2012	37	165.57	–	Čechová et al. (2017)
The Netherlands	2011–2014	120	42.68	–	
Norway	2001–2006	388	74.00	–	
Denmark	2011–2014	438	–	57.81–967.48	Antignac et al. (2016)
Finland		22	–	44.42–190.7	
France		96	–	14.26–397.27	
Russia	1997–2009	155	–	19–655	Mamontova et al. (2017)
Ghana	2014–2016	128	3.64	<LOQ-29.20	Asamoah et al. (2018)
China	2011	1760	6.6	2.3–19.0	Deng et al. (2012)
Northern Tanzania	2012	95	–	<LOQ-157.0	Müller et al. (2017)

–No data

Four PCBs were detected most frequently: PCB52, PCB101, PCB138, and PCB180 (Figure S2) were found in 34%, 6%, 83%, and 45% of samples, respectively.

**Fig. 1:**
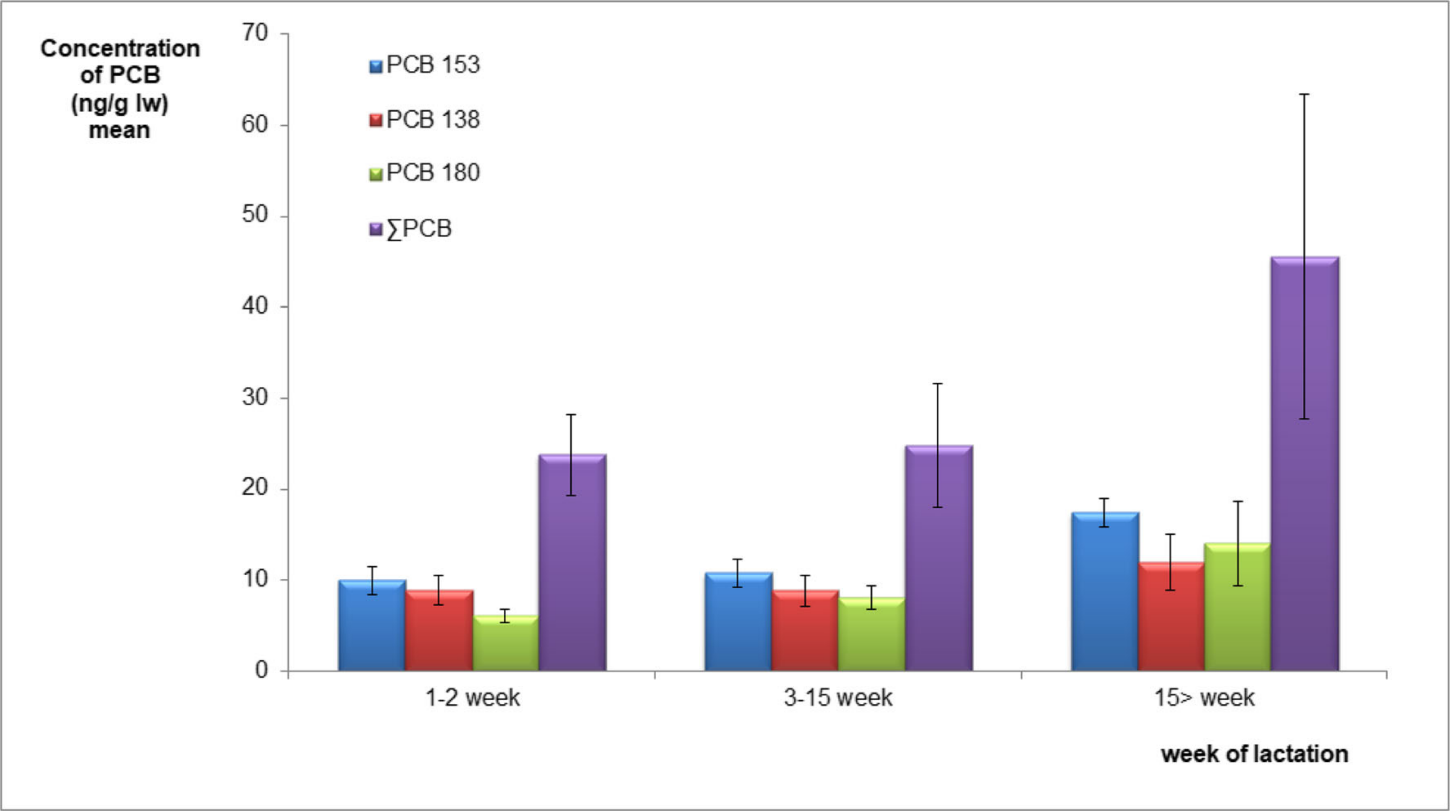
Mean concentrations of PCBs detected in milk samples, divided according to the week of lactation

This result confirmed that PCBs with six and more chlorine atoms are more resistant to metabolism, and that these congeners have strong affinity to accumulating in milk. The mean concentration of individual PCBs was the highest for PCB153, and the latter was the most abundant congener in the breast milk samples, followed by PCB180 > 52 > 138 > 101. Positive correlation was found between PCB153, 138, 180, *∑*PCB, and the week of lactation (Fig. 1; Table 3). Moreover, PCB153 was negatively correlated with dry weight and lipid content and consequently with energy value, whereas PCB180 was negatively correlated with protein content (Table 3).

**Table 3:** Correlations between PCBs and other measured variables

	Correlation matrix					
	Marked correlations are significant at *p* < 0.05000, *n* = 28					
	PCB 52	PCB101	PCB153	PCB138	PCB180	Sum of PCB
Lipids (g/100 mL)	− 0.124	0.132	− 0.560	− 0.201	− 0.353	− 0.351
Lactation period	0.317	0.182	0.633	0.525	0.796	0.662
Total protein (g/100 mL)	− 0.215	− 0.049	− 0.310	− 0.073	− 0.415	− 0.303
Nutritional value (g/100 mL)	− 0.182	− 0.059	− 0.294	− 0.053	− 0.409	− 0.282
Carbohydrates (g/100 mL)	0.014	0.017	0.070	0.097	0.341	0.154
Dry weight (g/100 mL)	− 0.166	0.087	− 0.512	− 0.133	− 0.287	− 0.315
Energy value (kcal/100 mL)	− 0.169	0.089	− 0.554	− 0.166	− 0.332	− 0.350

**Fig. 2:**
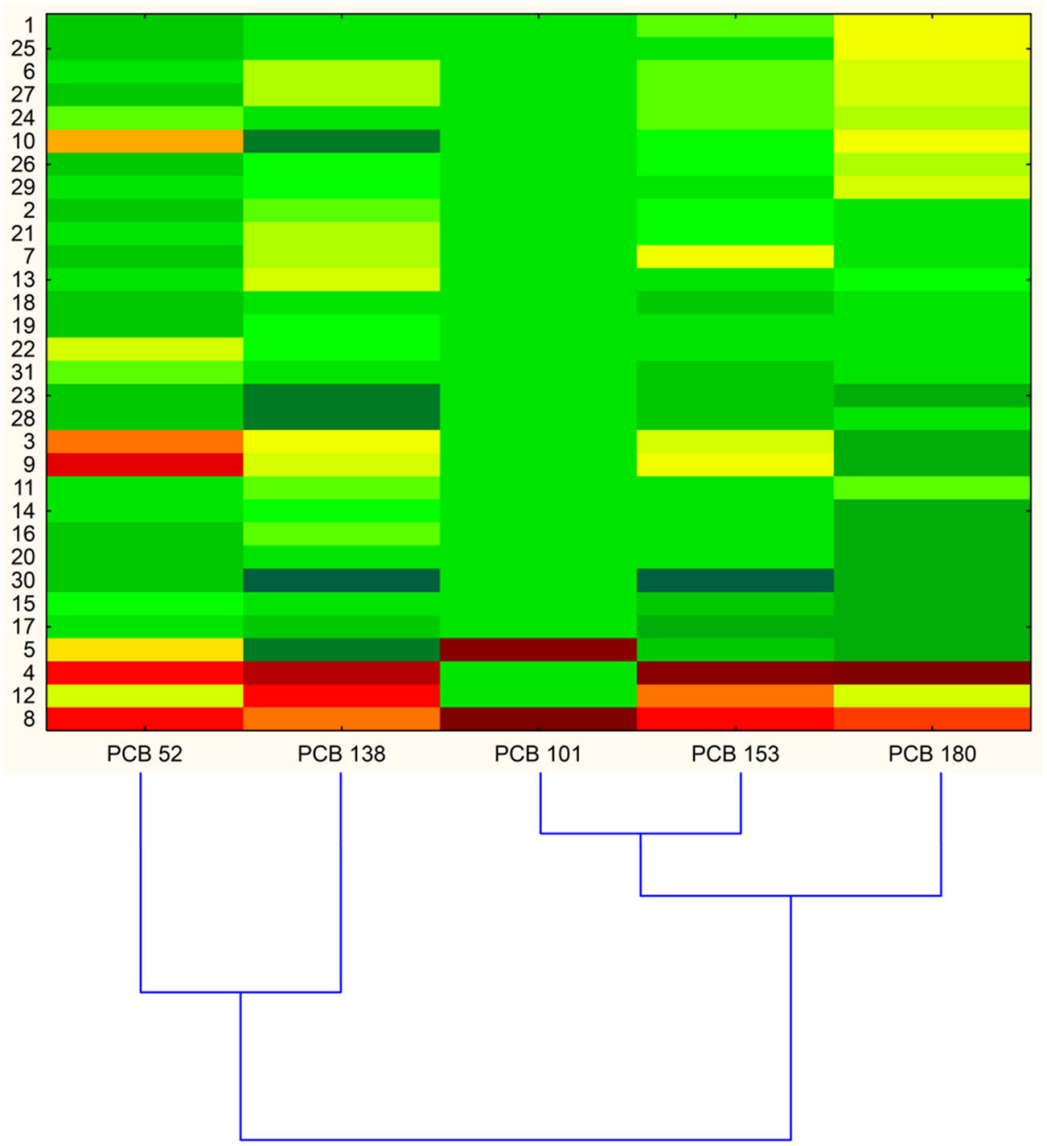
Cluster analysis heat map of investigated samples

Heat plot (Fig. 2) shows that congeners 153 and 180 create one cluster; both were identified in the largest number of samples tested, with average concentrations 13.08 and 11.52 ng/g, respectively. The highest concentrations were determined in sample no. 4 (38.82 ng/g and 33.16 ng/g). Their presence in the tested milk samples confirms their high tendency to bioaccumulate in tissues. Interestingly, as regards PCBs substituted with 6 chlorine atoms, PCB138 with its average concentration of 9.95 ng/g is significantly different from PCB153; however, the half-life of PCB138 is about 12 years, while that of PCB153 is 17 years.

Factor analysis (Table S3) revealed three latent variables with an Eigenvalue greater than 1 that explain more than 80% of cumulative variance. The first factor was positively loaded with congeners 153 and 138, while the second one with PCB52 and PCB101. Factor 3 was correlated with the last PCB—PCB180.

**Fig. 3:**
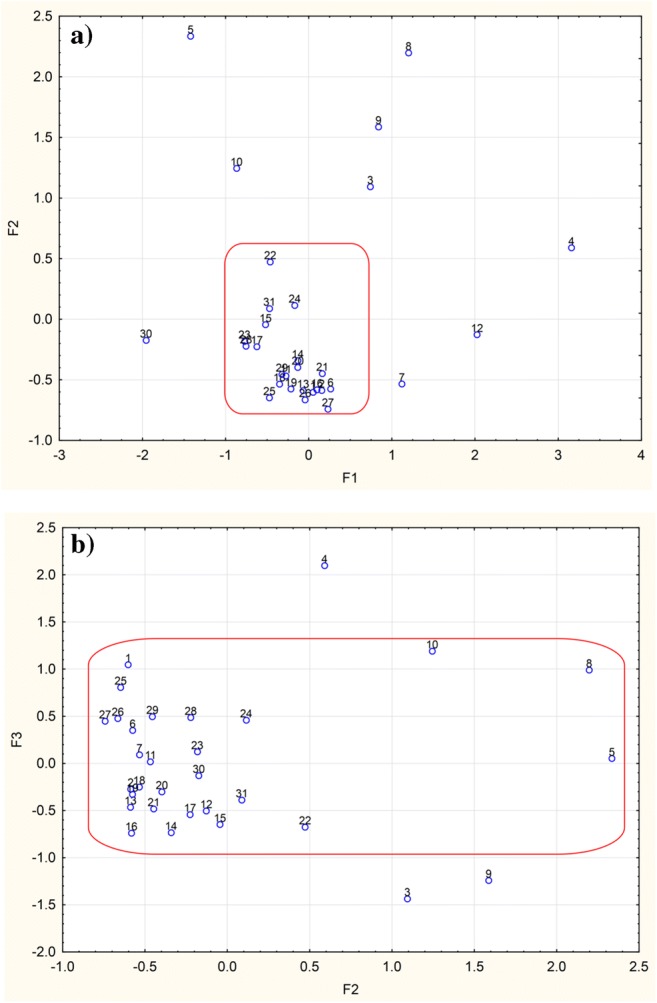
Classification of samples in the space of three latent factors: the first two factors (**a**) and third factor (**b**)

Figure 3a, b show the classification of investigated samples by factor analysis. In the case of the first two factors (Fig. 3a), the majority of milk samples that appeared outside the red zone were collected during early lactation period (samples: no. 5, week 1; no. 10, week 8, no. 3, week 2; no. 9, week 3; and no. 12, week 10) and the donors were under 30 years of age. It explains the presence of PCB52, which is less lipophilic and in consequence susceptible to faster elimination from the human body. In the case of samples no. 8 and no. 4, the lactation week was 36 and 44, respectively, and those women were older than 30. In these samples, the highest *∑*PCB content was found. One should pay attention to samples no. 4 and no. 30, which are found at the opposite sides of F1 axis. In the first one, the highest concentration of PCBs was measured while none of the investigated compounds was found in the second one. Sample no. 30 was collected in the 15th week of lactation from a 28-year-old mother after her third delivery. Assuming that this was her third breastfeeding, any contamination in the form of PCBs may have been removed during the first two feeding periods.

Taking into consideration the third factor (Fig. 3b), sample no. 4 contained the highest concentration of the sum of PCBs as well as the highest concentration of PCB180. PCB180 is composed of two phenyl rings with seven chlorine atoms. Such an amount of substituted chlorine atoms makes it highly resistant to degradation and elimination from the body. This sample also comes from the latest lactation period (week 44), from a mother after the second childbirth. On the other hand, in samples no. 3 and no. 9 (the 2nd and 3rd lactation periods, respectively), PCB52 (with 4 chlorine atoms) was found while PCB180 was absent; the women were first-time mothers. The obtained data confirms that PCBs with fewer chlorine atoms are removed from the body first.

To fully understand the obtained results, more data are required, including BMI before pregnancy and mother’s diet, as lack of such information can lead to misinterpretations. Parallel studies investigating the biological characteristics of the samples are essential. Investigating the correlation between the content of the studied compounds and human parameters of a given sample requires much more information about daily behavior as such data are very sensitive to even minor changes in human behavior and diet (Fernández-Cruz et al. 2017). The fact that PCB153 is the congener most often detected in the samples is in agreement with the results from other similar studies (Čechová et al. 2017; Klinčić et al. 2016; Müller et al. 2017; Szyrwińska and Lulek 2007). Consequently, PCB153 is considered as a marker of the presence of these organic impurities. In turn, Asamoah et al. (2018) showed that in Ghana, where contact with electronic waste (e-waste) is a significant exposure factor, the presence of PCB18 and PCB28 was dominant compared with other polychlorinated biphenyls. The reason is that old electronic devices tend to contain PCBs with fewer chlorine atoms attached to biphenyl rings. Another reason would be that chemical degradation results in degradation of higher chlorinated PCBs into less-toxic low-chlorinated ones (Takasuga et al. 2006). Furthermore, varying degree of milk contamination by PCBs may be caused by differences in diet and environment (Černá et al. 2010; Deng et al. 2012; Schuhmacher et al. 2007).

### Infant risk assessment

The exposure of breast-fed babies to toxic substances in milk is usually calculated from residue levels detected in human milk (Asamoah et al. 2018; Klinčić et al. 2016; Van Oostdam et al. 1999) (Eq. 2). Estimated daily intake of PCBs was lower than the tolerable daily intake. It confirmed that the analyzed human milk is safe for the infants. The results are presented in Table 4. The maximum value (*∑*PCBs = 0.47 μg/kg bw/day) of estimated daily intake for detected PCBs is less than 1.00 (reference value, Van Oostdam et al. 1999); this confirmed that feeding with this milk poses low risk to infants. However, taking into account the resistance of these compounds to degradation and the possibility of children being exposed to them, biomonitoring is recommended, so such tests should be systematically carried out. These investigations can minimize the risk of immune system problems, asthma, allergy, or neurological disorders. An example of a monitoring program involving a long-term cross-sectional research is Czech human biomonitoring project, started in1996 (Komprda et al. 2019).

**Table 4:** Mean and range of estimated daily intake of PCBs for the analyzed milk (μg/kg body weight/day)

PCBs	Mean	Median	Minimum	Maximum	Reference^a^
52	0.05	0.06	0.02	0.09	1.00
101	0.04	0.04	0.03	0.04	
153	0.06	0.05	0.03	0.15	
138	0.04	0.04	0.03	0.10	
180	0.05	0.04	0.02	0.13	
*∑*PCBs	0.14	0.12	0.04	0.47	

^a^Van Oostdam et al. (1999)

## Conclusions

Thirty-one breast milk samples were collected from Polish mothers living in Kuyavian-Pomeranian region. We found significant positive correlation between the concentrations of three congeners detected in abundance (nos. 153, 138, and 180) as well as the sum of detected indicators (*∑*PCB). The conducted research confirmed that lack of such information about donors as, e.g., BMI, before pregnancy or diet style limits the interpretation of the obtained data. However, the collected information confirms that environmental pollutants such as polychlorinated biphenyls are still present in the world around us and consequently in human milk. It is important that the current concentration is much lower than in previous years and that infants’ actual daily intake does not exceed the tolerable daily intake. However, it is recommended to repeat such tests regularly; a modified QuEChERS method with GC-MS can be a fast tool for tracking impurities in breast milk. Due to many possibilities of changing the individual steps of the QuEChERS method, researchers can still improve this method by using different sorbents and reagents to eliminate co-extractive compounds.

## Electronic supplementary material

ESM 1 (DOCX 817 kb)
