# Supplementary material for: QuEChERS extraction coupled to GC-MS for a fast determination of polychlorinated biphenyls in breast milk from Polish women
Source: Environ Sci Pollut Res Int. 2019 Aug 26;26(30):30988–99. doi: 10.1007/s11356-019-06201-y (PMC6828831; doi:10.1007/s11356-019-06201-y)
Supplement: Supplementary file 1 — (DOCX 817 kb) [file 11356_2019_6201_MOESM1_ESM.docx]

Supplementary material for:

**QuEChERS extraction coupled to GC-MS for a fast determination of polychlorinated biphenyls in breast milk from Polish women**

Martyna Pajewska-Szmyt^1,2^, Elena Sinkiewicz-Darol^3,4^, Urszula Bernatowicz-Łojko^3,4^, Tomasz Kowalkowski^1,2^, Renata Gadzała-Kopciuch^1,2,*^, Bogusław Buszewski^1,2^

^1)^Department of Environmental Chemistry and Bioanalytics, Faculty of Chemistry,

Nicolaus Copernicus University in Toruń, 7 Gagarin St., 87-100 Toruń, Poland

^2)^Interdisciplinary Centre for Modern Technologies, Nicolaus Copernicus University,
4 Wileńska St, PL-87100 Toruń, Poland

^3)^Ludwik Rydygier Provincial Polyclinic Hospital in Toruń, Human Milk Bank, Św. Józefa 53-59, 87-100 Toruń, Poland

^4)^Human Milk Bank Foundation, 128J Podkowy St., 04-937 Warsaw, Poland

*Corresponding author: Renata Gadzała-Kopciuch (rgadz@umk.pl)

**3 Tables**

**2 Figure**

Table S1. Examples of studies where QuEChERS method was used for PCB determination.

| **PCBs congener** | **Matrices** | **Clean-up stage** | **Recovery (%)** | **Reference** |
| --- | --- | --- | --- | --- |
| 28, 52, 101, 118, 153, 138, 180 | Fish tissues (tilapia, salmon) | MgSO_4_, PSA | 26-98 | Norli et al. 2011 |
| 25,52, 101, 81, 77, 123, 118, 114, 153, 105, 138, 126, 167, 156, 157, 180, 169, 189 | **Human milk** |  | 89-103 | Luzardo et al. 2013 |
| 28, 52, 101, 118, 138,153,150 | Meat products (salami, soudjouk, sausage) |  | 95.7-101 | Kuzukiran et al. 2016 |
| 1, 18, 29, 31, 5, 77, 50, 52, 44, 66, 1047, 28, 153, 154, 141, 138, 187, 183 | Mussel (Mytilus sp.) |  | 79-115 | Madureira et al. 2014 |
| 28, 52, 101, 118, 153, 138, 180 | fish | ZrO_2_@Fe_3_O_4_  Fe_3_O_4_-OPA (n-octadecylphosphonic acid) | 71-115 | Peng et al. 2015 |
| 28, 52, 1101, 118, 153, 138, 180 | Fish muscle  **Human milk** | Captiva ND lipid cartidge  MgSO_4_, Z-Sep | 89-96  71-102 | Baduel et al. 2015 |
| 28,52, 101, 118, 138,153, 180 | White sturgeon (liver and gonad tissues) | MgSO_4_, PSA, C_18_  Performed twice | 60-103 | Morrison et al. 2016 |
| 77, 81, 105, 114, 123, 118, 126, 156, 157, 167, 169, 170, 180, 189 | Kale, pork, salmon, avocado | EMR-Lipid (enhanced matrix removal of lipids) | 45-102 | Han et al. 2016 |
| 1, 5, 18, 31, 52, 44, 66, 101, 87, 110, 151, 153, 141, 137, 187, 183, 180, 170, 206 | Catfish tissue | MgSO_4_, PSA, C_18_ | 71-102 | Chamkasem et al. 2016 |
| 18, 31, 28, 52, 44, 70, 81, 101, 123, 118, 114, 105, 126, 149,153, 138, 167, 156, 157, 169, 180, 189 | Honey | PSA, C_18_ | 50-99 | Al-Alam et al. 2017 |
| 18, 28, 52, 101, 138, 153, 180 | **Human milk** | MgSO_4_, PSA, C_18_ | 82-110 | Asamoah et al. 2018 |

Table S2. General information on the breast milk donors and particular milk samples.

| Sample no. | Living area | Age | Pregnancy | Childbirth | Lactation  period (week) | Lipid  (g/100mL) | Total protein (g/100mL) | Nuritional  protein (g/100mL) | Carbohydrates  (g/100 mL) | Dry weight (g/100 mL) | Energy value (kcal/100 mL) |
| --- | --- | --- | --- | --- | --- | --- | --- | --- | --- | --- | --- |
| 1 | Rypin | 28 | 1 | 1 | 7 | 2.8 | 1.3 | 1.0 | 7.6 | 11.9 | 62 |
| 2 | Toruń | 30 | 1 | 1 | 8 | 3.9 | 1.6 | 1.3 | 7.5 | 13.2 | 73 |
| 3 | Gdańsk | 28 | 2 | 1 | 2 | 3.8 | 2.0 | 1.7 | 6.9 | 13.0 | 71 |
| 4 | Bydgoszcz | 33 | 2 | 2 | 44 | 2.8 | 1.0 | 0.8 | 7.6 | 11.6 | 61 |
| 5 | Bydgoszcz | 26 | 2 | 1 | 1 | 4.7 | 1.6 | 1.2 | 7.3 | 13.8 | 79 |
| 6 | Gdańsk | 29 | 2 | 1 | 2 | 4.7 | 1.4 | 11 | 7.5 | 13.8 | 80 |
| 7 | Toruń | - | - | - | - | 3.8 | 0.7 | - | 6.9 | - | - |
| 8 | Toruń | 31 | 2 | 1 | 36 | 3.3 | 1.1 | 0.9 | 7.6 | 12.2 | 65 |
| 9 | Toruń | 26 | 1 | 1 | 3 | 3.1 | 1.0 | 0.8 | 7.2 | 11.5 | 62 |
| 10 | Toruń | 28 | 2 | 2 | 8 | 3.6 | 1.3 | 1.1 | 7.8 | 12.9 | 70 |
| 11 | Aleksandrów Kujawski | 28 | 1 | 1 | 15 | 3.9 | 1.4 | 1.1 | 7.8 | 13.3 | 73 |
| 12 | Kowalewo Pomorskie | 29 | 1 | 1 | 10 | 3.8 | 0.9 | 0.7 | 7.2 | 12.2 | 68 |
| 13 | Runowo | 30 | 2 | 2 | 7 | 3.3 | 1.4 | 1.1 | 7.8 | 12.7 | 68 |
| 14 | Brześć Kujawski | 31 | 2 | 2 | 6 | 5.1 | 1.4 | 1.2 | 7.3 | 14.1 | 83 |
| 15 | Bydgoszcz | - | - | - | - | 1.8 | 2.3 | 1.9 | 7.5 | 11.8 | 56 |
| 16 | Toruń | 25 | 1 | 1 | 3 | 4.0 | 1.9 | 1.5 | 7.4 | 13.5 | 75 |
| 17 | Toruń | 27 | 2 | 2 | 10 | 3.6 | 1.3 | 1.0 | 7.4 | 12.6 | 69 |
| 18 | - | 26 | 1 | 1 | 17 | 4.3 | 1.2 | 1.0 | 7.4 | 13.1 | 76 |
| 19 | - | 21 | 1 | 1 | 3 | 3.4 | 1.6 | 1.3 | 7.5 | 12.6 | 68 |
| 20 | - | 26 | 1 | 1 | 1 | 3.9 | 1.9 | 1.5 | 7.1 | 13.0 | 72 |
| 21 | - | 38 | 3 | 1 | 1 | 2.8 | 2.9 | 2.4 | 6.5 | 12.4 | 65 |
| 22 | Bydgoszcz | - | - | - | - | - | - | - | - | - | - |
| 23 | - | 29 | 2 | 2 | 3 | 2.5 | 0.8 | 0.7 | 5.6 | 9.1 | 49 |
| 24 | Bydgoszcz | 30 | 2 | 2 | 36 | 1.7 | 0.9 | 0.7 | 7.9 | 10.6 | 51 |
| 25 | Toruń | 23 | 1 | 1 | 40 | 2.9 | 0.9 | 0.7 | 7.9 | 11.7 | 62 |
| 26 | Toruń | 27 | 3 | 2 | 15 | 3.6 | 1 | 0.8 | 7.9 | 12.7 | 69 |
| 27 | Toruń | 33 | 2 | 2 | 11 | 3.7 | 1.3 | 1 | 7.6 | 12.9 | 71 |
| 28 | Toruń | 36 | 2 | 2 | 15 | 1.5 | 1.1 | 0.8 | 7.8 | 10.6 | 50 |
| 29 | Toruń | 27 | 1 | 1 | 36 | 2.2 | 1.3 | 1 | 7.8 | 11.5 | 58 |
| 30 | Toruń | 28 | 3 | 3 | 15 | 3.6 | 1.2 | 0.9 | 7.8 | 12.8 | 69 |
| 31 | Toruń | 31 | 2 | 2 | 5 | 4.2 | 1.9 | 1.5 | 7.9 | 14.2 | 79 |

- no data

Table S3. Factor loadings.

|  | F1 | F2 | F3 |
| --- | --- | --- | --- |
| PCB52 | 0.46 | **0.88** | 0.09 |
| PCB 101 | -0.02 | **0.72** | 0.19 |
| PCB 153 | **0.94** | 0.33 | 0.42 |
| PCB 138 | **0.95** | 0.12 | 0.09 |
| PCB 180 | 0.62 | 0.13 | **0.83** |


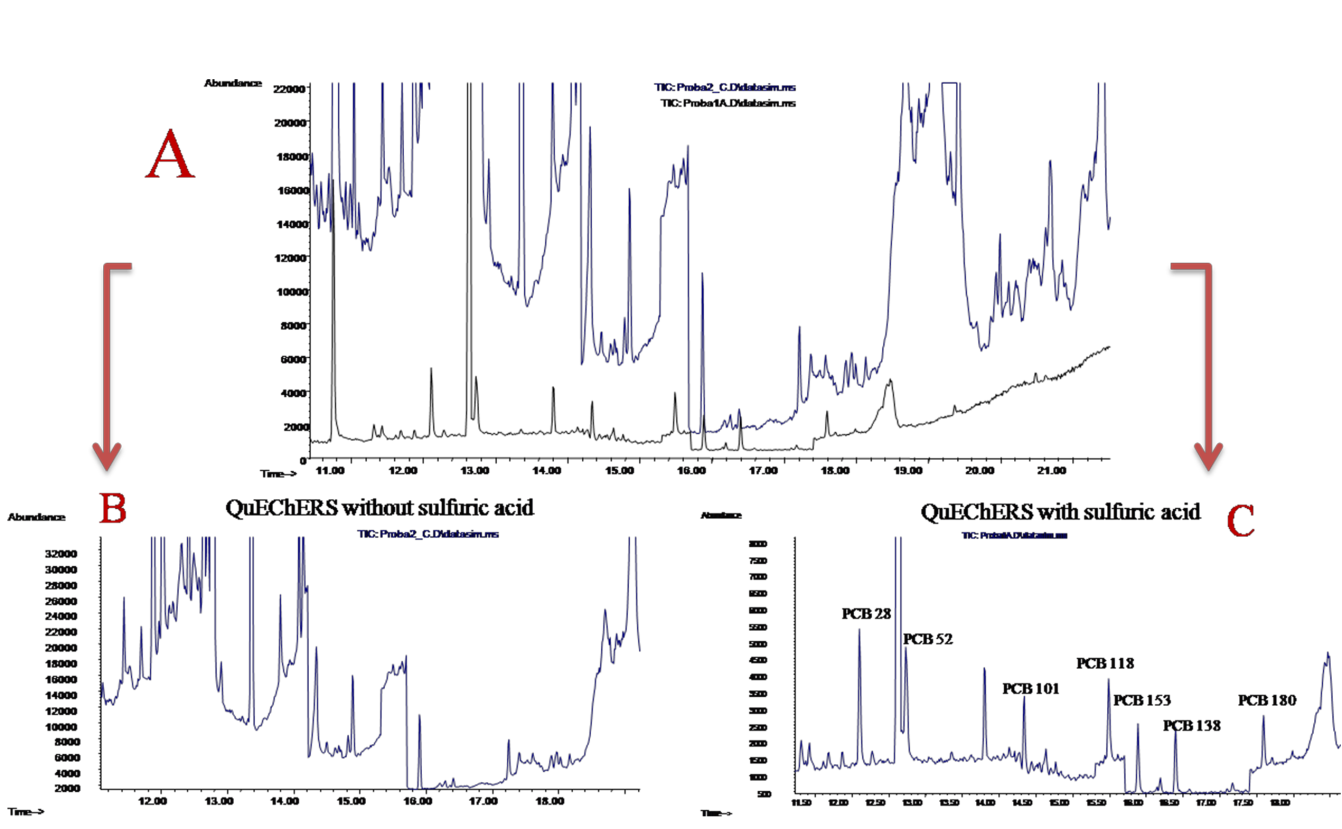


Figure S1. Chromatograms of spiked samples (10 ng/mL of each PCB); QuEChERS method without sulfuric acid addition for lipid removal (blue line - A and B) and with sulfuric acid addition (black line – A and blue line – C).


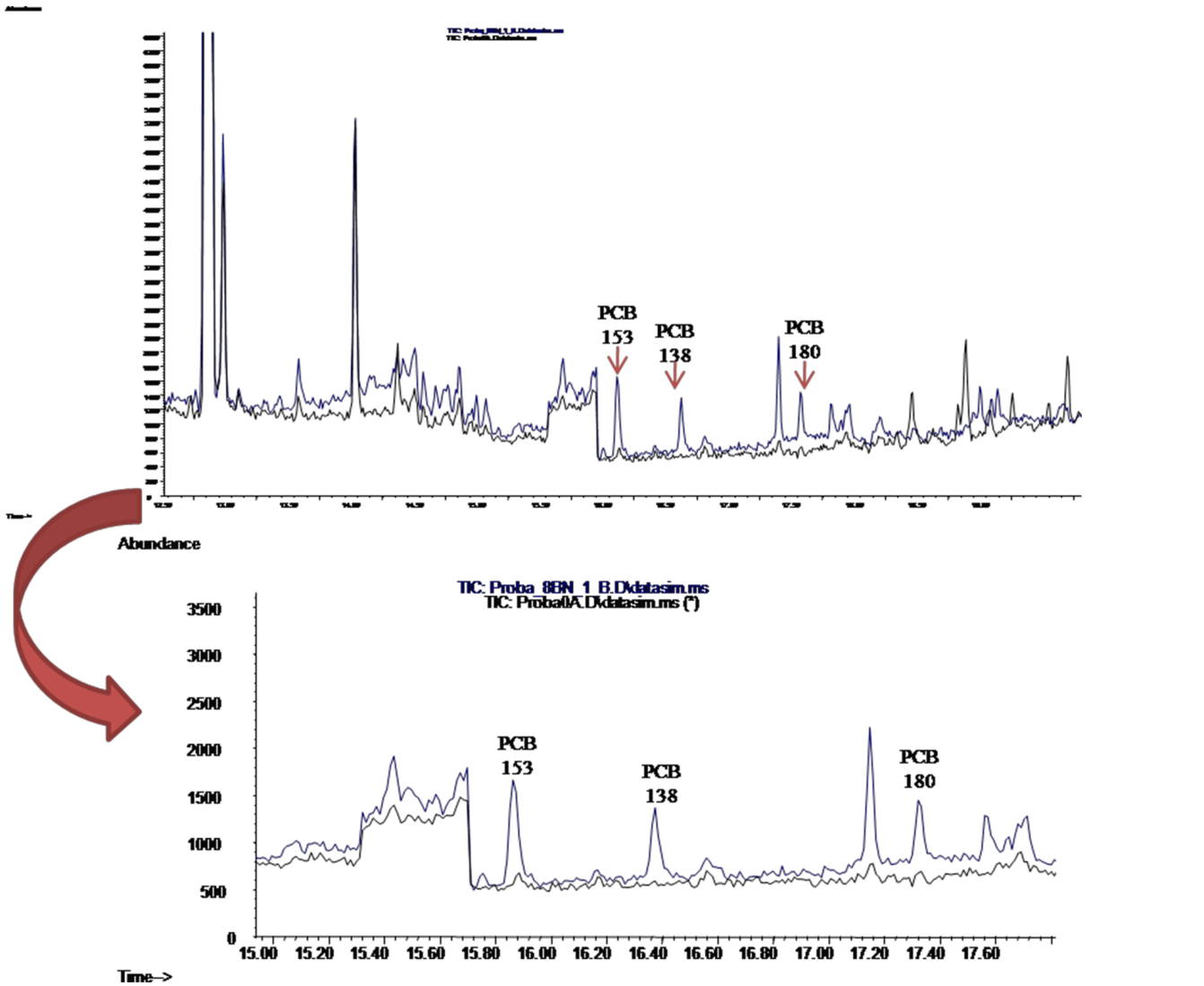


Figure S2. Example of a chromatogram of breast milk samples (blue line) with detected PCB 153, 138, and 180 and of the blank sample used as quality control (black line).

**Reference**

Al-Alam J, Fajloun Z, Chbani A, Millet M (2017) A multiresidue method for the analysis of 90 pesticides, 16 PAHs, and 22 PCBs in honey using QuEChERS-SPME. Anal Bioanal Chem 409:5157-5169. https://doi.org/10.1007/s00216-017-0463-y.

Asamoah A, Essumang DK, Muff J, Kucheryavskiy SV, Søgaard EG (2018) Assessment of PCBs and exposure risk to infants in breast milk of primiparae and multiparae mothers in an electronic waste hot spot and non-hot spot areas in Ghana. Sci Total Environ 612:1473–1479. https://doi.org/10.1016/j.scitotenv.2017.08.177

Baduel C, Mueller JF, Tsai H, Gomez Ramos MJ (2015) Development of sample extraction and clean-up strategies for target and non-target analysis of environmental contaminants in biological matrices. J Chromatogr A 1426:33–47. https://doi.org/10.1016/j.chroma.2015.11.040

Chamkasem N, Lee S, Harmon T (2016) Analysis of 19 PCB congeners in catfish tissue using a modified QuEChERS method with GC-MS/MS. Food Chem 192:900–906. https://doi.org/10.1016/j.foodchem.2015.07.088

Han L, Matarrita J, Sapozhnikova Y, Lehotay SJ (2016) Evaluation of a recent product to remove lipids and other matrix co-extractives in the analysis of pesticide residues and environmental contaminants in foods. J Chromatogr A 1449:17-29. https://doi.org/10.1016/j.chroma.2016.04.052.

Kuzukiran O, Filazi A (2016) Determination of selected polychlorinated biphenyl residues in meat products by QuEChERS method coupled with gas chromatography-mass spectrometry. Food Anal. Methods 9(7), 1867-1875. https://doi.org/10.1007/s12161-015-0367-4

Luzardo OP, Ruiz-Suárez N, Almeida-González M, Henríquez-Hernández LA, Zumbado M, Boada LD (2013) Multi-residue method for the determination of 57 Persistent Organic Pollutants in human milk and colostrum using a QuEChERS-based extraction procedure. Anal Bioanal Chem 405:9523–9536.
https://doi.org/10.1007/s00216-013-7377-0

Madureira TV, Santos C, Velhote S, Cruzeiro C, Rocha E, Rocha MJ (2014) Contamination levels of polychlorinated biphenyls in wild versus cultivated samples of female and male mussels (Mytilus sp.) from the Northwest Coast of Iberian Penisula - new application for QuEChERS (Quick, Easy. Cheap, Effective, Rugged, and Safe) methodology. Environ Sci Pollut Res Int 21(2):1528-1540. https://doi.org/10.1007/s11356-013-2017-y

Morrison SA, Sieve KK, Ratajczak RE, Bringolf RB, Belden JB (2016) Simultaneous extraction and cleanup of high-lipid organs from white sturgeon (Acipenser transmontanus) for multiple legacy and emerging organic contaminants using QuEChERS sample preparation. Talanta 146:16–22. https://doi.org/10.1016/j.talanta.2015.08.021

Norli HR, Christiansen A, Deribe E (2011) Application of QuEChERS method for extraction of selected persistent organic pollutants in fish tissue and analysis by gas chromatography mass spectrometry. J Chromatogr A 1218(41):7234-7241. https://doi.org/10.1016/j.chroma.2011.08.050.

Peng XT, Jiang L, Gong Y, Hu XZ, Peng LJ, Feng YQ (2015) Preparation of mesoporous ZrO2-coated magnetic microsphere and its application in the multiresidue analysis of pesticides and PCB in fish by GC-MS/MS. Talanta 132:118-125. https://doi.org/10.1016/j.talanta.2014.08.069.
